# Adaptiveness of RGB-image derived algorithms in the measurement of fractional vegetation coverage

**DOI:** 10.1186/s12859-022-04886-6

**Published:** 2022-08-30

**Authors:** Chuangye Song, Jiawen Sang, Lin Zhang, Huiming Liu, Dongxiu Wu, Weiying Yuan, Chong Huang

**Affiliations:** 1 State Key Laboratory of Vegetation and Environmental Change, Institute of Botany, Chinese Academy of Sciences, Beijing, 100093 China; 2 University of Chinese Academy of Sciences, Beijing, 100049 China; 3 Satellite Application Centre for Ecology and Environment, Ministry of Ecology and Environment, Beijing, 100094 China; 4 State Key Laboratory of Resources and Environmental Information System, Institute of Geographic and Natural Resources Research, Chinese Academy of Sciences, Beijing, 100101 China

**Keywords:** Digital image, Grassland, Field survey, Mobile smart phone, Canopy density

## Abstract

**Background:**

Fractional vegetation coverage (FVC) is a crucial parameter in determining vegetation structure. Automatic measurement of FVC using digital images captured by mobile smart devices is a potential direction for future research on field survey methods in plant ecology, and this algorithm is crucial for accurate FVC measurement. However, there is a lack of insight into the influence of illumination on the accuracy of FVC measurements. Therefore, the main objective of this research is to assess the adaptiveness and performance of different algorithms under varying light conditions for FVC measurements and to deepen our understanding of the influence of illumination on FVC measurement.

**Methods and results:**

Based on a literature survey, we selected four algorithms that have been reported to have high accuracy in automatic FVC measurements. The first algorithm (Fun01) identifies green plants based on the combination of $R/G$, $B/G$, and $ExG$ ($R$, $G$, and $B$ are the actual pixel digital numbers from the images based on each RGB channel, $ExG$ is the abbreviation of the Excess Green index), the second algorithm (Fun02) is a decision tree that uses color properties to discriminate plants from the background, the third algorithm (Fun03) uses $ExG-ExR$ ($ExR$ is the abbreviation of the Excess Red index) to recognize plants in the image, and the fourth algorithm (Fun04) uses $ExG$ and $O{\text{tsu}}$ to separate the plants from the background. $Otsu$ is an algorithm used to determine a threshold to transform the image into a binary image for the vegetation and background. We measured the FVC of several surveyed quadrats using these four algorithms under three scenarios, namely overcast sky, solar forenoon, and solar noon. FVC values obtained using the Photoshop-assisted manual identification method were used as a reference to assess the accuracy of the four algorithms selected. Results indicate that under the overcast sky scenario, Fun01 was more accurate than the other algorithms and the MAPE (mean absolute percentage error), BIAS, relBIAS (relative BIAS), RMSE (root mean square error), and relRMSE (relative RMSE) are 8.68%, 1.3, 3.97, 3.13, and 12.33%, respectively. Under the scenario of the solar forenoon, Fun02 (decision tree) was more accurate than other algorithms, and the MAPE, BIAS, relBIAS, RMSE, and relRMSE are 22.70%, − 2.86, − 7.70, 5.00, and 41.23%. Under the solar noon scenario, Fun02 was also more accurate than the other algorithms, and the MAPE, BIAS, relBIAS, RMSE, and relRMSE are 20.60%, − 6.39, − 20.67, 7.30, and 24.49%, respectively.

**Conclusions:**

Given that each algorithm has its own optimal application scenario, among the four algorithms selected, Fun01 (the combination of $R/G$, $B/G$, and $ExG$) can be recommended for measuring FVC on cloudy days. Fun02 (decision tree) is more suitable for measuring the FVC on sunny days. However, it considerably underestimates the FVC in most cases. We expect the findings of this study to serve as a useful reference for automatic vegetation cover measurements.

**Supplementary Information:**

The online version contains supplementary material available at 10.1186/s12859-022-04886-6.

## Background

Fractional vegetation coverage (FVC) is defined as the vertical projection of the crown and shoot area on a horizontal surface and is expressed as a fraction or percentage of the reference area [1]. FVC is an important parameter of the vegetation structure. Variations in FVC reflect changes in the ecological environment and play an important role in indicating changes in regional and global land-cover as well as landscape differentiation.

Ground FVC measurements are usually performed visually [2] with the accuracy depending on the person conducting the measurement, which can cause bias and inconsistency between observers. Sykes et al. [3] and Chen et al. [4] evaluated the accuracy of a visual method and found that the relative error ranged from 10 to 40% [3, 4] and it can be difficult for an observer to distinguish between cover intervals of < 10% [5]. With the development of photography-related technologies, digital imaging technologies have presented an alternative approach to accurately measure FVC. Over the last decade, the emergence of mobile smart devices such as iPhones has made FVC measurement more convenient and efficient. Mobile smart devices can be used to capture vegetation photos and record important spatial and environmental information, such as the latitude, longitude, altitude, humidity, temperature, shooting direction, and the horizontal tilt angle. Based on embedded algorithms, mobile smart devices can process the images collected and automatically measure the FVC with high efficiency. Therefore, using mobile smart devices to capture vegetation photographs and the measurement of FVC has promising prospects.

The algorithm is essential in the use of mobile smart devices for effectively measuring FVC. Algorithms used for FVC measurement can be divided into two categories: one is the threshold-based algorithm which functions according to color or vegetation indices [6–11], such as the color index of vegetation extraction ($CIVE$) [12], the excess green index ($ExG$) [13], excess green minus excess red ($ExG-ExR$) [14], and many other indices [15]. The other is cluster analysis based on training samples and object-based image analysis methodology [16–20]. Among these two categories, threshold-based algorithms are often recommended for being embedded in a mobile smart device to perform automatic FVC measurements because of their appropriate calculation quantities and lack of human–computer interactions [21]. The influence of light conditions on the performance of threshold-based algorithms has been assessed in plant identification and FVC measurements. Campbell [22] found that the color properties of vegetation and color-based indices are not sensitive to variations in illumination, indicating that they possess considerable potential for the identification of plant components. Lati et al. [23] also concluded that indices such as $R/G$, $B/G$, and $ExG$ are insensitive to variations in illumination. However, color-indice-based algorithms have been found to be easily influenced by the intensity of the illumination and the light angle. Lukina et al. [24] concluded that a high level of reflection and shadows impact the accuracy of FVC measurements, and the best moment for shooting is when the sun reaches a location with a high solar elevation. Meyer et al. [25] suggested that a disproportionate red color originating from different light sources could obscure the color properties of digital images, making it more difficult to identify green plants using RGB-based indices. Booth et al. [26] showed that the highlighted areas in photos have a considerable influence on FVC measurements. Chen et al. [4] found that the FVC being measured decreased with an increase in the illumination intensity. According to Sadeghi-Tehran et al. [20], portions of the canopy that are shadowed or have a high specular reflectance can strongly contribute to the underestimation of vegetation pixels within an image. Although numerous studies have been conducted on the influence of illumination on FVC measurement, there has been considerable debate around this issue, and no firm conclusions have been reached from the findings of previous studies. Therefore, it is necessary to assess the adaptiveness of different algorithms under varying light conditions to deepen our understanding of the influence of illumination on FVC measurement.

In this study, we selected four RGB-color-based algorithms that have been reported to have a high level of accuracy in FVC measurement and assessed their adaptiveness and performance under different application scenarios. This aim is to provide useful information for the selection of an appropriate algorithm under a specific application scenario for FVC measurements. We believe that the findings of this study will provide scientific support for performing FVC measurements using mobile phones.

## Data and methods

### Experiment design

**Fig. 1:**
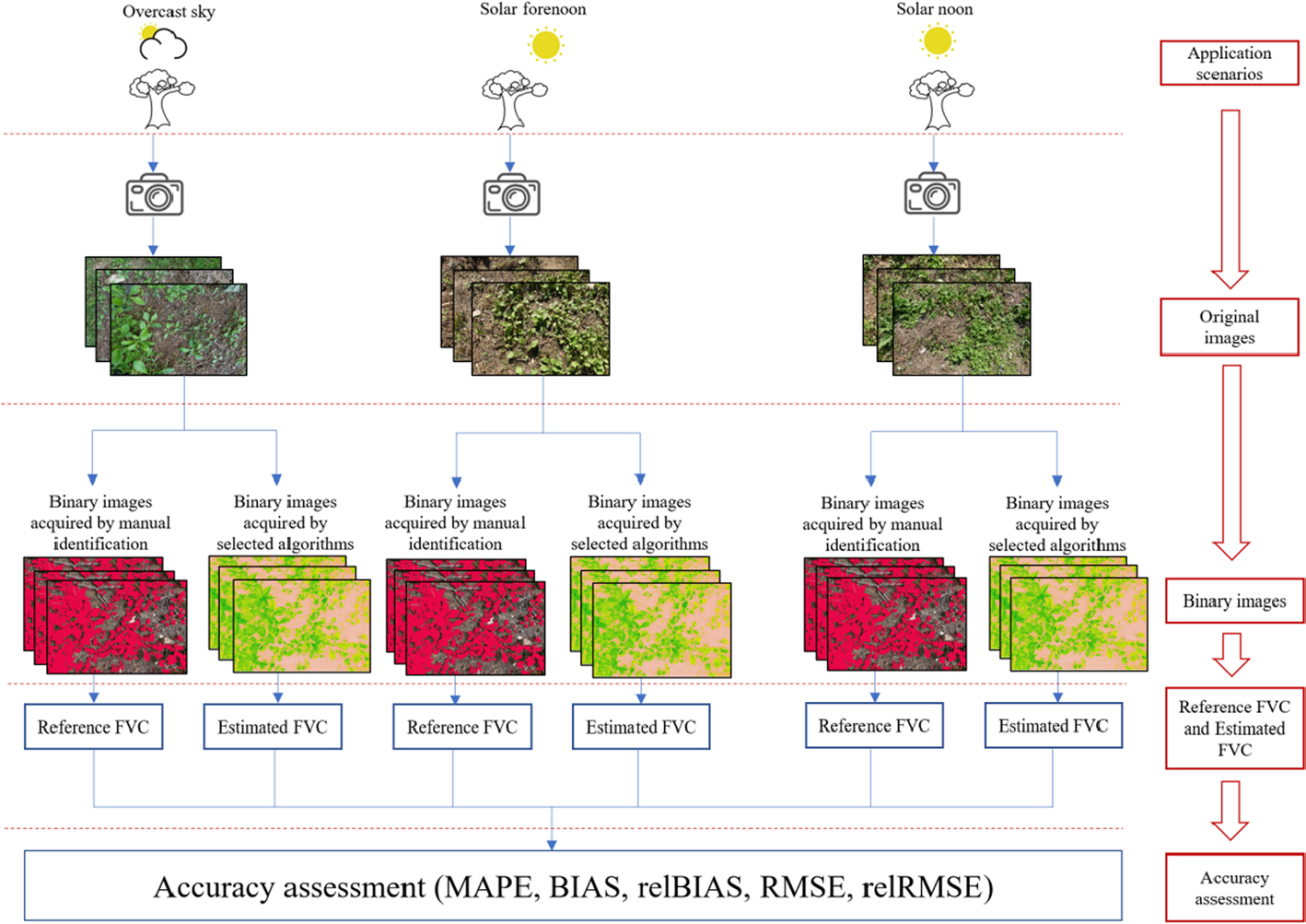
Schematic diagram of the fractional vegetation coverage (FVC) measurement and accuracy assessment in our research

The first step in our research was to capture downward-looking vegetation photos under three application scenarios, namely overcast sky, solar forenoon, and solar noon (for details, see “Application scenarios and photo collection” section) using a digital camera. The photos acquired were then processed using the four selected algorithms (“Selected algorithms of FVC measurement” section) to separate the plants from the background and to obtain binary images of vegetation and non-vegetation. The FVC was then obtained as the ratio of the vegetation pixel number to the total number of pixels. Adobe Photoshop software was used to manually process these photos to obtain the FVC values (“Reference FVC” section). These values were used as references to assess the accuracy of the FVC values measured by the four algorithms selected (“Accuracy assessment” section). The flowchart of the study is shown in Fig. 1.

### Application scenarios and photo collection

The vegetation images were acquired under three application scenarios, namely overcast sky, when the percentage of clouds in the sky was higher than 70%; solar forenoon, approximately 9 AM, when the percentage of clouds in the sky was lower than 10%; and solar noon, approximately 12 AM, when the cloud percentage was lower than 10%. Under the solar forenoon and solar noon scenarios, the sun was occasionally obscured by clouds, and the images used in this study were obtained when the clouds moved away, and sunlight was present on the leaves. The sun was generally obscured by clouds during the experiment when shooting images under the cloudy sky scenario, and the light that illuminated the leaves was scattered.

The experiment was conducted at the Beijing Botanical Garden, Chinese Academy of Sciences, during early October 2020. The dominant plant taxa were *Hydrocotyle sibthorpioides*, *Digitaria sanguinalis*, *Oxalis corniculata*, *Rumex acetosa*, *Viola philippica*, *Chloris virgata*, *Setaria viridis*, *Polygonum aviculare*, *Inula japonica*, *Convolvulus arvensis*, *Taraxacum mongolicum*, *Trigonotis peduncularis*, *Plantago asiatica*, and *Ixeris polycephala*.

Nadir images were acquired using a SONY DSC-RX100M5A digital camera under three scenarios of overcast sky, solar forenoon, and solar noon. The camera was set to operate in automatic mode using the highest image resolution (5472 × 3648 pixels). The spatial resolution of the images with this digital camera mounted 1 m above the plant targets translated to 350 pixels/in.

Under the overcast sky, solar forenoon, and solar noon scenarios, 34, 30, and 30 digital images, respectively, were taken and processed to assess the accuracy of each algorithm. Some of these images are presented in Additional file 1: Fig. S1.

### Selected algorithms of FVC measurement

The FVC is calculated as the ratio of the number of vegetation pixels to the total number of pixels in the image.

$$FVC = X_{{\text{i}}} /X_{j} \times 100$$

where $X_{{\text{i}}}$ is the number of vegetation pixels and $X_{{\text{j}}}$ is the total number of pixels in the image.

The vegetation pixels were identified using the following four algorithms where$,R$,$G$, and $B$ are the actual pixel digital number (DN) values in the red, green, and blue channels, respectively.

1. Fun01 was designed to identify the green vegetation components using the following criteria [8]:
  $$R/G < P1,\,\,B/G < P2\quad and\quad 2G - R - B > P3$$
  where *P1* and *P2* are assigned a value near 1 [27], and *P3* typically has a value of approximately 20 to differentiate the green vegetation from the background [28, 29]. The parameter values used by Patrignani et al. are *P1* = 0.95, *P2* = 0.95, and *P3* = 20 and we used the same parameter settings [8].
  Fun01 is based on the union of $R/G$, $B/G$ and the excess green index. $R/G$, $B/G$, and the excess green index ($ExG$) have been proven to be effective in the identification of green plants [28, 29].
2. Fun02 can identify green components, colored flowers, and other non-vegetation components. It identifies pixels that satisfy the following rules [7]:
  $G>R>B$, green leaves, peak green leaves, and olivine leaves.
  $G>B>R$, blackish-green leaves, reflective leaves, or light-colored leaves.
  $B>G>R$, cyan stones or blue stones.
  $R>G>B$ and $\left|R\left.-B\right|\le 10\right.$, yellow leaves.
  $R>G>B$ and $\left|R\left.-B\right|>10\right.$, soil, dead wood, or dead leaves.
  $R>B>G$ and $(R-B)>40$ and $(R-G)>40$, red flowers or red leaves.
  $R>B>G$ and $\left|R\left.-B\right|<40\right.$ and $\left|R\left.-G\right|<40\right.$, soil.
  $B>R>G$, blue or purple flowers.
  Fun02 can be used to identify vegetation and non-vegetation components based on the combination of DN values of the red, green, and blue channels. Theoretically, green plants, colored flowers, and other non-vegetation components can be identified using this algorithm.
3. Fun03 recognizes the vegetation components based on the excess green index ($ExG$) and the excess red index ($ExR$) with the following criteria for recognition [28]:
  $$ExG-ExR>0$$
  where $$ExG=2\times G-B-R$$, $$ExR=1.4\times R-G$$
  The $ExG-ExR$ vegetation index does not require a special threshold calculation. Pixels with positive values are defined as vegetation components, and pixels with negative values are defined as non-vegetation components. Therefore, $ExG-ExR$ can self-generate a binary image with a constant threshold of zero [28].
4. Fun04 is based on the $ExG$ and Otsu algorithms [30]. The Otsu method offers an optimal index thresholding value by maximizing the between-class tonal variance and minimizing the within-class tonal variance of the image [28]. This was used to achieve a threshold value to segment the $ExG$ image into the vegetation components and the background.

### Reference FVC

The FVC reference values for the color images were obtained using Adobe Photoshop (Version 13.0) software. The vegetation region was carefully selected using a mouse and the Photoshop “magic wand tool”. We then used the Photoshop “brush tool” to paint the selected vegetation region in red color. We also used “the brush tool” to paint the plants in areas of shadow, and the tiny plant areas that were excluded when using the “magic wand tool.” When all the plant pixels had been selected and painted in red, we defined the vegetation pixels as 1 and the non-vegetation pixels as 0. We then transformed the original image into a binary image, and obtained the number of plant pixels in the “histogram”. Finally, the FVC was calculated as the percentage of pixels classified as plant components.

### Accuracy assessment

Linear regression was used to detect the relationship between the measured and reference FVC values. We then compared the FVC values measured with the reference FVC values using analysis of variance to determine whether there are significant differences.

To evaluate the accuracy of measurement, we first calculated the relative error (RE) of the FVC values measured using the algorithms selected and then analyzed the distribution of RE. We also calculated the mean absolute percentage error (MAPE), BIAS, the root mean square error (RMSE), the relative BIAS (relBIAS), and the relative RMSE (relRMSE) to evaluate the measurement accuracy. The RE, MAPE, BIAS, relBIAS, RMSE, and relRMSE were defined using the following equations:

$$RE=\frac{{x}_{i}-{x}_{ir}}{{x}_{ir}}\times 100$$

$$\mathrm{MAPE}=\sum\limits_{i=1}^{n}\left(\frac{\left|{x}_{i}-{x}_{ir}\right|}{{x}_{ir}}\times 100\right)/n$$

$${\text{BIAS}} = \frac{{\sum\nolimits_{{i = 1}}^{n} {(x_{i} - x_{{ir}} )} }}{n}$$

$$\mathrm{relBIAS}=\frac{\sum_{i=1}^{n}(\frac{{x}_{i}}{{x}_{ir}}-1)}{n}\times 100$$

$$\mathrm{RMSE}=\sqrt{\frac{\sum_{i=1}^{n}{\left({x}_{i}-{x}_{ir}\right)}^{2}}{n}}$$

$$\mathrm{relRMSE}=\sqrt{\frac{\sum_{i=1}^{n}{\left(\frac{{x}_{i}}{{x}_{ir}}-1\right)}^{2}}{n}}\times 100$$

Here $, {x}_{i}$ is the ith measurement, ${x}_{ir}$ is the ith reference, and *n* is the number of measurements.

## Results

### Overcast sky scenario

**Fig. 2:**
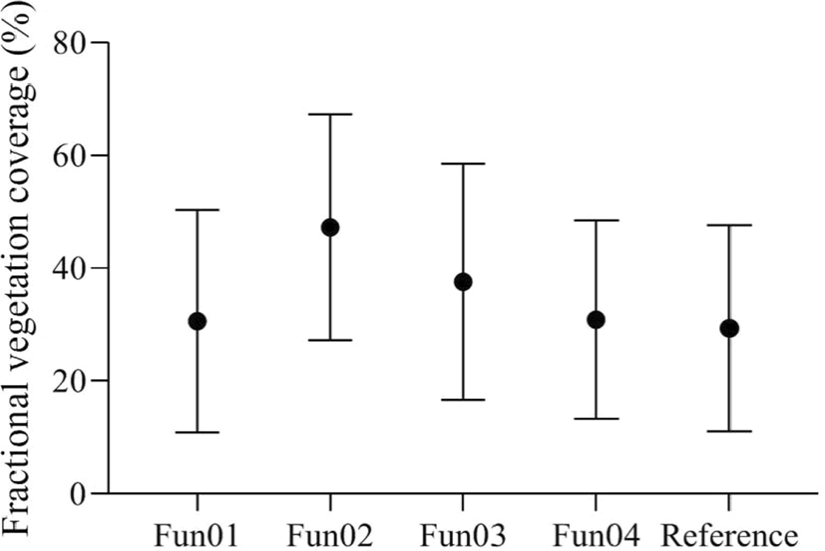
The mean fractional vegetation coverage values measured using different algorithms and the reference values under the overcast sky scenario. Fun01 is based on the combination of $R/G$, $B/G$ and $2G-R-B$. Fun02 is the decision tree algorithm based on the RGB color. Fun03 uses the excess green and red indices to recognize the plants in the image. Fun04 uses the excess green index and the Otsu algorithm to identify the plants in the image

The mean FVC values measured using the algorithms selected under the overcast sky scenario are shown in Fig. 2. The analysis of variance indicated that there were no significant differences between the FVC values measured using Fun01, Fun03, Fun04, and the reference FVC values (*p* = 0.779, *p* = 0.08, *p* = 0.735, respectively), whereas Fun02 significantly overestimated the FVC (*p* = 0.000).

**Fig. 3:**
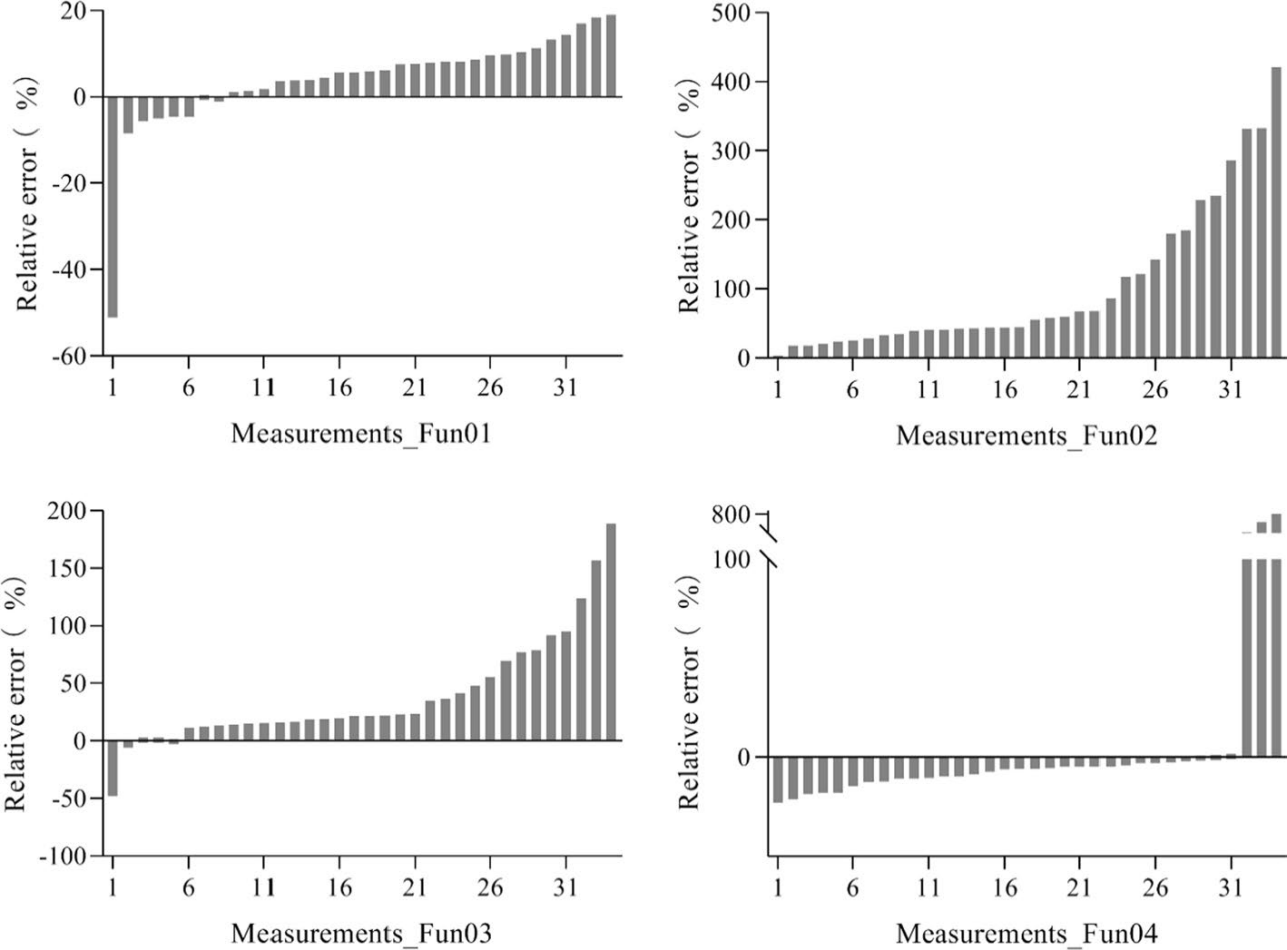
The relative error of the fractional vegetation cover measured using different algorithms under the overcast sky scenario. Fun01 is based on the combination of $R/G$, $B/G$ and $2G-R-B$. Fun02 is the decision tree algorithm based on the RGB color. Fun03 uses the excess green and red indices to recognize the plants in the image. Fun04 uses the excess green index and the Otsu algorithm to identify the plants in the image

Figure 3 depicts the RE of the four algorithms. Among the 34 Fun01-measured FVC values (34 measurements), the RE of more than 80% of the measurements was lower than 10% including both positive and negative RE. The RE of only one measurement was higher than 20%. For Fun02, the RE of the three measurements was lower than 10% and the RE of twenty-nine measurements was higher than 50%. For Fun03, the RE for 44% of the measurements was lower than 20%, the RE of four measurements was lower than 10%, and the RE of 40% of the measurements was higher than 30%. For Fun04, the RE of the 60% measurements was lower than 10%, the RE of the 90% measurements was lower than 25%, and the RE of the three measurements was higher than 100%. It was also found that Fun01, Fun02, and Fun03 overestimated the FVC in most measurements, while Fun04 underestimated the FVC for most measurements.

**Fig. 4:**
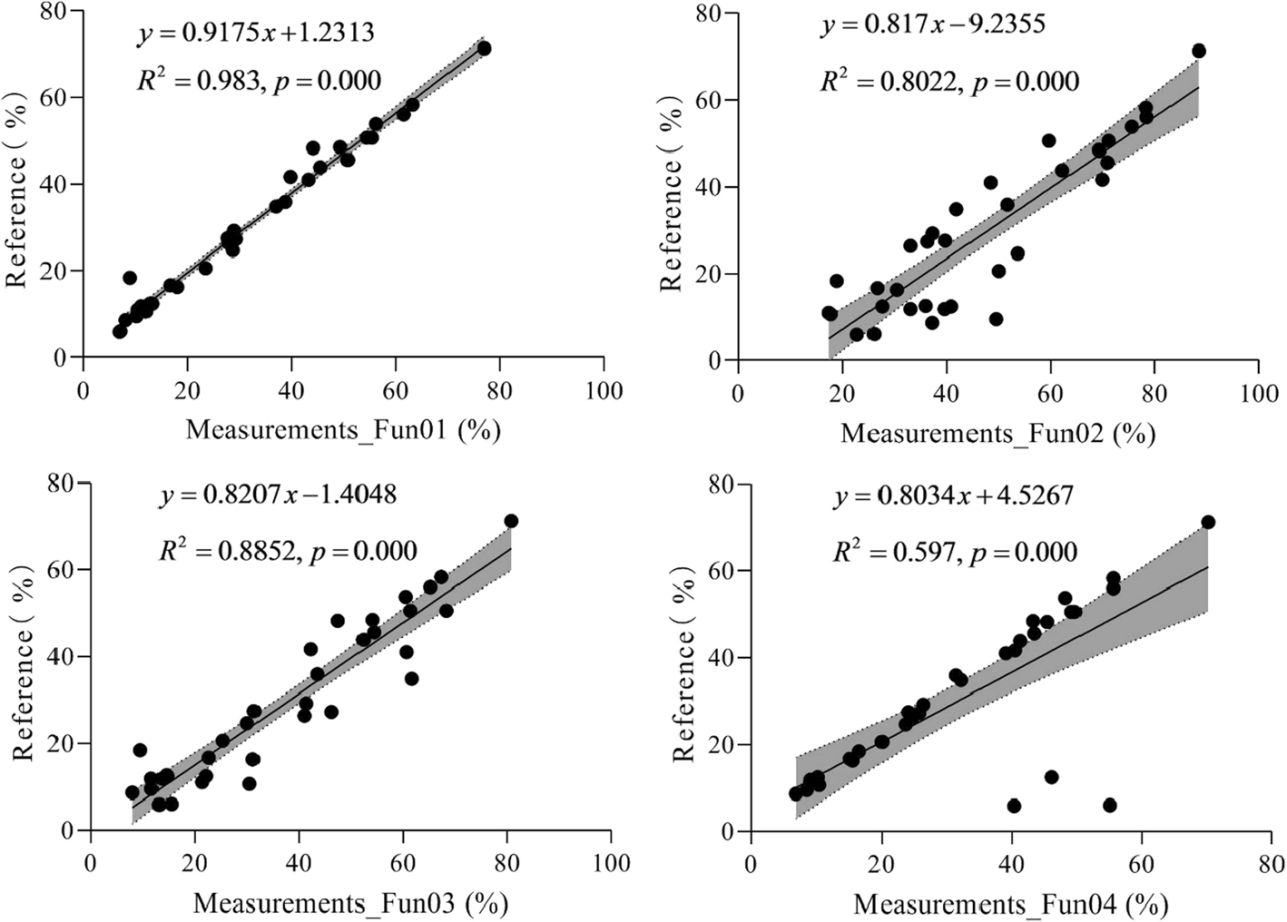
The linear relation between the fractional vegetation cover measured and the reference under the overcast sky scenario. Shaded areas represent the 95% confidence intervals for the regression lines. Fun01 is based on the combination of $R/G$, $B/G$ and $2G-R-B$. Fun02 is the decision tree algorithm based on the RGB color. Fun03 uses the excess green and red indices to recognize the plants in the image. Fun04 uses the excess green index and the Otsu algorithm to identify the plants in the image

Figure 4 shows the linear relationship between the measured and reference FVC values. From Fig. 4, we can see that the R^2^ of Fun01 is higher than that of the other algorithms, followed by Fun03 and Fun02. For Fun04, the RE of the three measurements was higher than that of the other measurements (Fig. 3), which led to a low R^2^. If these three abnormal values were removed from the regression, the R^2^ increases to 0.995.

Table 1 lists the accuracy variables of the selected algorithms under the overcast sky scenario. The accuracy of Fun01 was higher than that of the other algorithms, whereas Fun02 had the lowest accuracy among the four algorithms. For Fun04, if the three abnormally measured FVC values were omitted from the assessment (Fig. 4), the MAPE, BIAS, relBIAS, RMSE, and relRMSE changed to 8.59, − 2.07, − 8.58, 2.42, and 10.53, respectively. The measurement accuracy of Fun04 is similar to that of Fun01.

**Table 1:** The measurement accuracy of different algorithms under the overcast sky scenario

	MAPE (%)	BIAS	relBIAS (%)	RMSE	relRMSE(%)
Fun01	8.68	1.3	3.97	3.13	12.33
Fun02	103.43	17.89	103.4	19.93	148.5
Fun03	42.31	8.14	38.94	10.82	61.14
Fun04	56.87	1.57	41.21	12.02	177.99

Fun01 is based on the combination of $R/G$, $B/G$ and $2G-R-B$. Fun02 is the decision tree algorithm based on the RGB color. Fun03 uses the excess green and red indices to recognize the plants in the image. Fun04 uses excess green index and Otsu algorithm to identify plants in the image

MAPE, mean absolute percentage error; relBIAS, relative BIAS; RMSE, root mean square error; relRMSE, relative root mean square error

In general, when the sky is overcast, Fun01 is more accurate than the other algorithms. The accuracy of Fun04 is also excellent when the three abnormal measurements are omitted from the assessment. Fun01, Fun02, and Fun03 tended to overestimate the FVC in the measurements, whereas Fun04 underestimated it in most measurements.

### Solar forenoon scenario

**Fig. 5:**
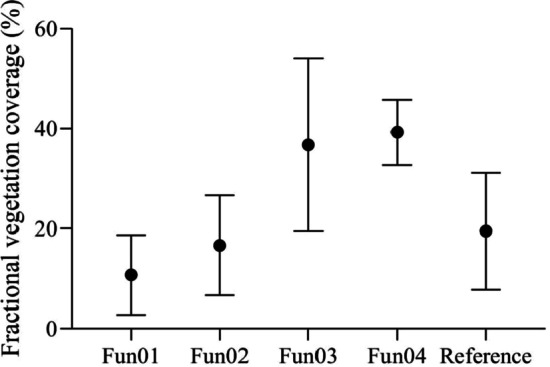
The mean fractional vegetation coverage values measured using different algorithms and the reference under the solar forenoon scenario. Fun01 is based on the combination of $R/G$, $B/G$ and $2G-R-B$. Fun02 is the decision tree algorithm based on the RGB color. Fun03 uses the excess green and red indices to recognize the plants in the image. Fun04 uses the excess green index and the Otsu algorithm to identify the plants in the image

The mean FVC values measured using the four algorithms under the solar forenoon scenario are shown in Fig. 5. The analysis of variance indicated that Fun01 significantly underestimated the FVC (*p* = 0.001), Fun03 and Fun04 significantly overestimated the FVC (*p* = 0.001), while there was no significant difference between the FVC values measured using Fun02 and the reference FVC values (*p* = 0.278).

**Fig. 6:**
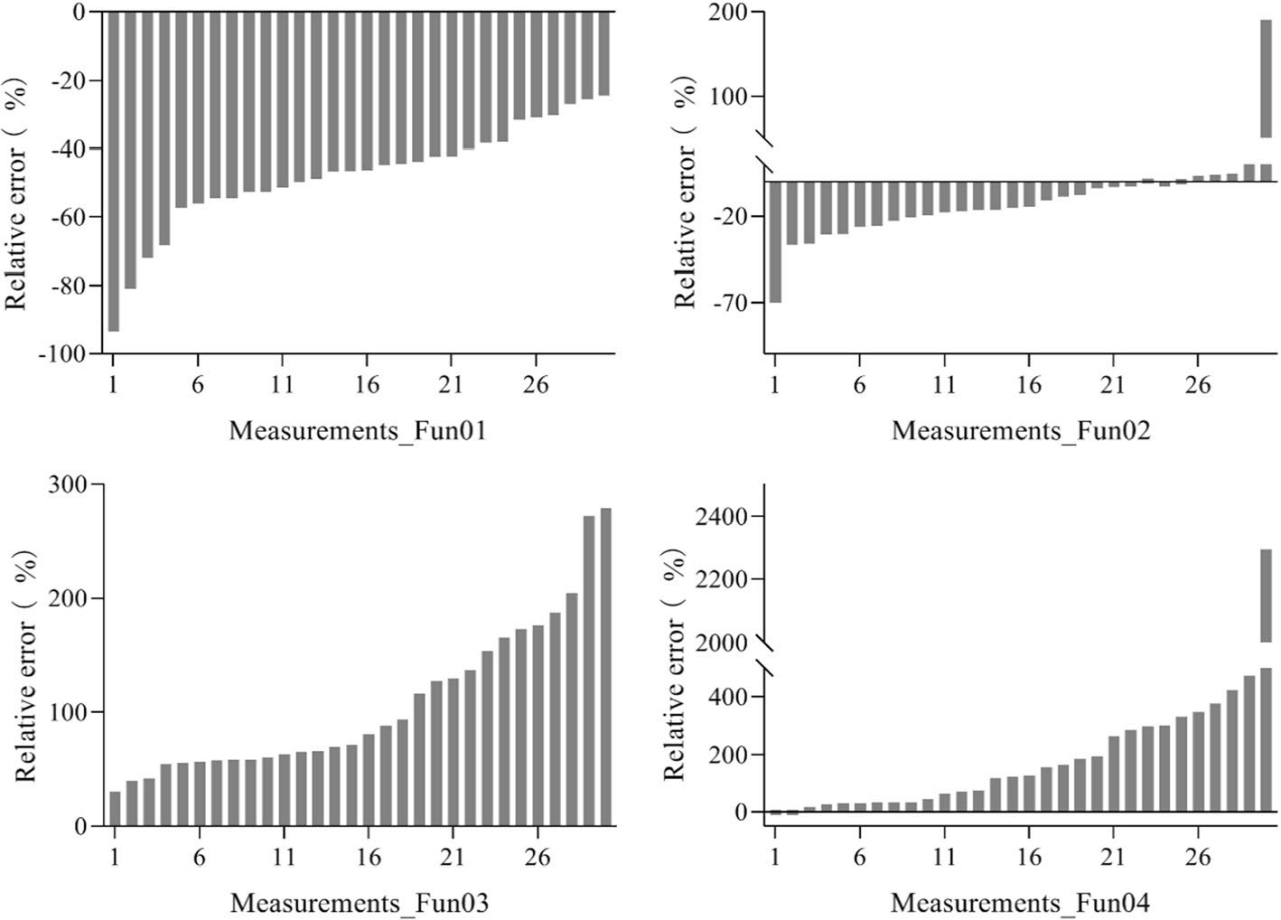
The relative error of the fractional vegetation coverage of different algorithms measured under the solar forenoon scenario. Fun01 is based on the combination of $R/G$, $B/G$ and $2G-R-B$. Fun02 is the decision tree algorithm based on the RGB color. Fun03 uses the excess green and red indices to recognize the plants in the image. Fun04 used the excess green index and the Otsu algorithm to identify the plants in the image

The RE of the four algorithms are shown in Fig. 6. Among the Fun01-measured FVC values (30 measurements), the RE for all the measurements was higher than 20%. For Fun02, the RE of 67% of the measurements was lower than 20% including both positive and negative RE. For Fun03, the RE of all the measurements was higher than 30%. For Fun04, the RE of only three measurements was lower than 20%. Fun01 and Fun02 underestimated the FVC in most measurements, while Fun03 and Fun04 overestimated the FVC in most measurements.

**Fig. 7:**
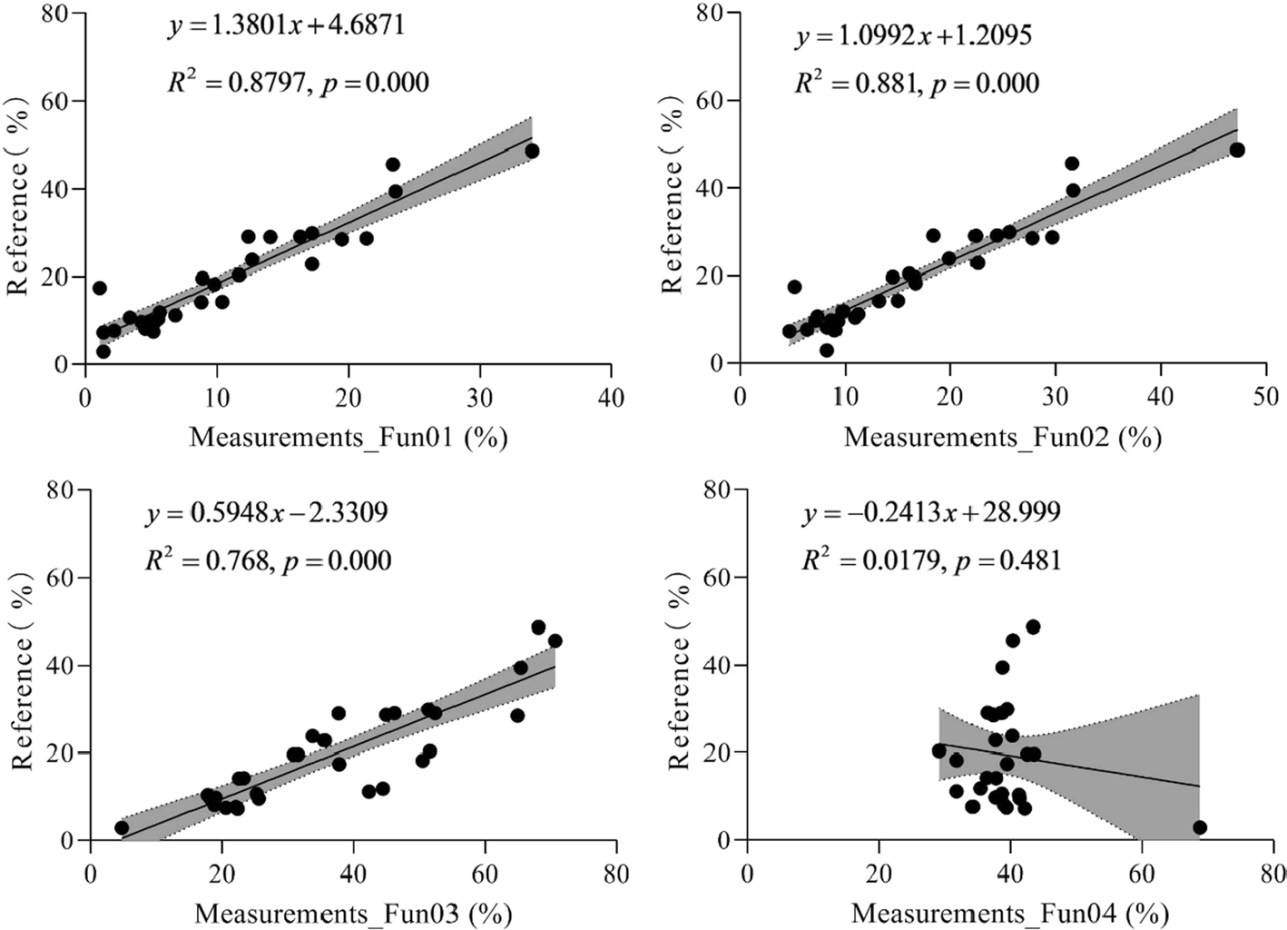
The linear relation between the fractional vegetation cover measured and the reference under the solar forenoon scenario. Shaded areas represent the 95% confidence intervals for the regression lines. Fun01 is based on the combination of $R/G$, $B/G$ and $2G-R-B$. Fun02 is the decision tree algorithm based on the RGB color. Fun03 uses the excess green and red indices to recognize the plants in the image. Fun04 uses the excess green index and the Otsu algorithm to identify the plants in the image

Figure 7 shows the linear relationship between the measured and reference FVC values. From Fig. 7, we can see that the R^2^ of Fun02 is higher than that of the other algorithms, followed by Fun01, Fun03, and Fun04.

Table 2 lists the accuracy variables for different algorithms under solar forenoons. We can see that the accuracy of Fun02 is higher than that of other algorithms, followed by Fun01 and Fun03, and the accuracy of Fun04 was the lowest among the four algorithms.

**Table 2:** Measurement accuracy of different algorithms under the solar forenoon scenario

	MAPE (%)	BIAS	relBIAS (%)	RMSE	relRMSE(%)
Fun01	47.80	− 8.77	− 48.00	10.09	50.33
Fun02	22.70	− 2.86	− 7.70	5.00	41.23
Fun03	108.1	1.88	108.00	19.36	126.75
Fun04	230.50	19.75	229.00	24.16	467.58

Fun01 is based on the combination of $R/G$, $B/G$ and $2G-R-B$. Fun02 is the decision tree algorithm based on the RGB color. Fun03 uses the excess green and red indices to recognize the plants in the image. Fun04 uses the excess green index and the Otsu algorithm to identify the plants in the image

MAPE, mean absolute percentage error; relBIAS, relative bias; RMSE, root mean square error; relRMSE, relative root mean square error

Under the solar forenoon scenario, the accuracy of Fun02 was higher than that of the other algorithms, with a mean RE of 23%. Fun02 tended to underestimate the FVC in most measurements.

### Solar noon scenario

**Fig. 8:**
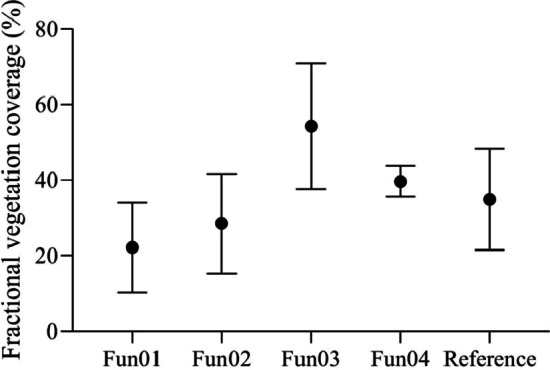
The mean fractional vegetation cover values measured using different algorithms and the reference fractional vegetation cover under the solar noon scenario. Fun01 is based on the combination of $R/G$, $B/G$ and $2G-R-B$. Fun02 is the decision tree algorithm based on the RGB color. Fun03 used the excess green and red indices to recognize the plants in the image. Fun04 uses the excess green index and the Otsu algorithm to identify the plants in the image

The mean FVC values measured using these algorithms under the solar noon scenario are shown in Fig. 8. The analysis of variance indicated that Fun01 significantly underestimated the FVC (*p* = 0.000). No significant difference was observed between the Fun02 and Fun04 estimated FVC values and the reference FVC values (*p* = 0.035, *p* = 0.114). Fun03 significantly overestimated the FVC values (*p* = 0.001).

**Fig. 9:**
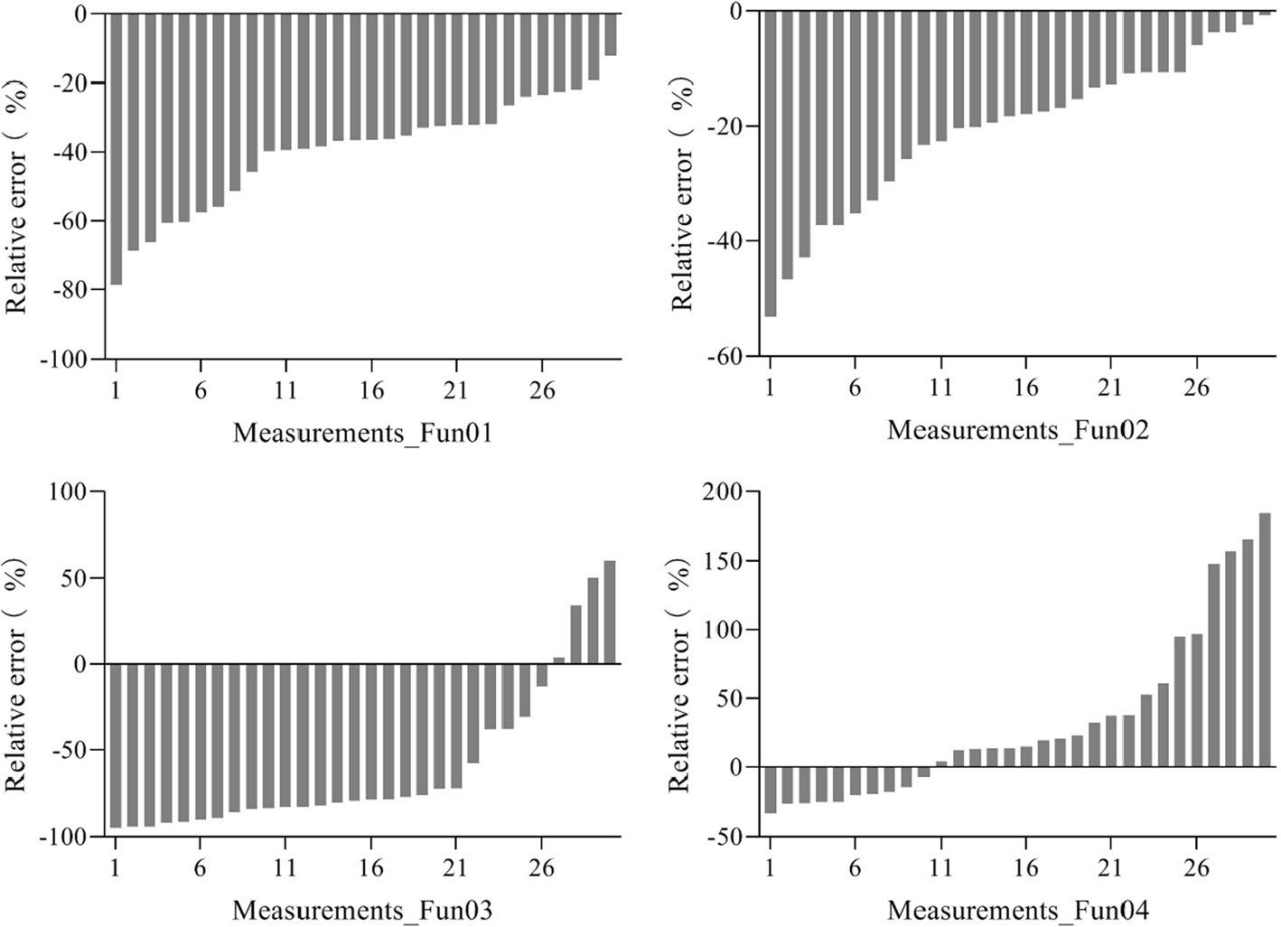
The relative error of the fractional vegetation cover measured using different algorithms under the solar noon scenario. Fun01 is based on the combination of $R/G$, $B/G$ and $2G-R-B$. Fun02 is the decision tree algorithm based on RGB color. Fun03 used the excess green and red indices to recognize the plants in the image. Fun04 uses the excess green index and the Otsu algorithm to identify the plants in the image

The RE of the four algorithms are shown in Fig. 9. Among the Fun01-measured FVC values (30 measurements), the RE for 90% of measurements was higher than 20%, and the RE of 80% of measurements was higher than 30%. For Fun02, the RE of 60% of the measurements was lower than 20%. For Fun03, the RE of 90% of the measurements was higher than 30%. For Fun04, the RE of the 40% measurement was lower than 20%. Fun01, Fun02, and Fun03 underestimated the FVC in most measurements, while Fun04 overestimated the FVC in most measurements.

**Fig. 10:**
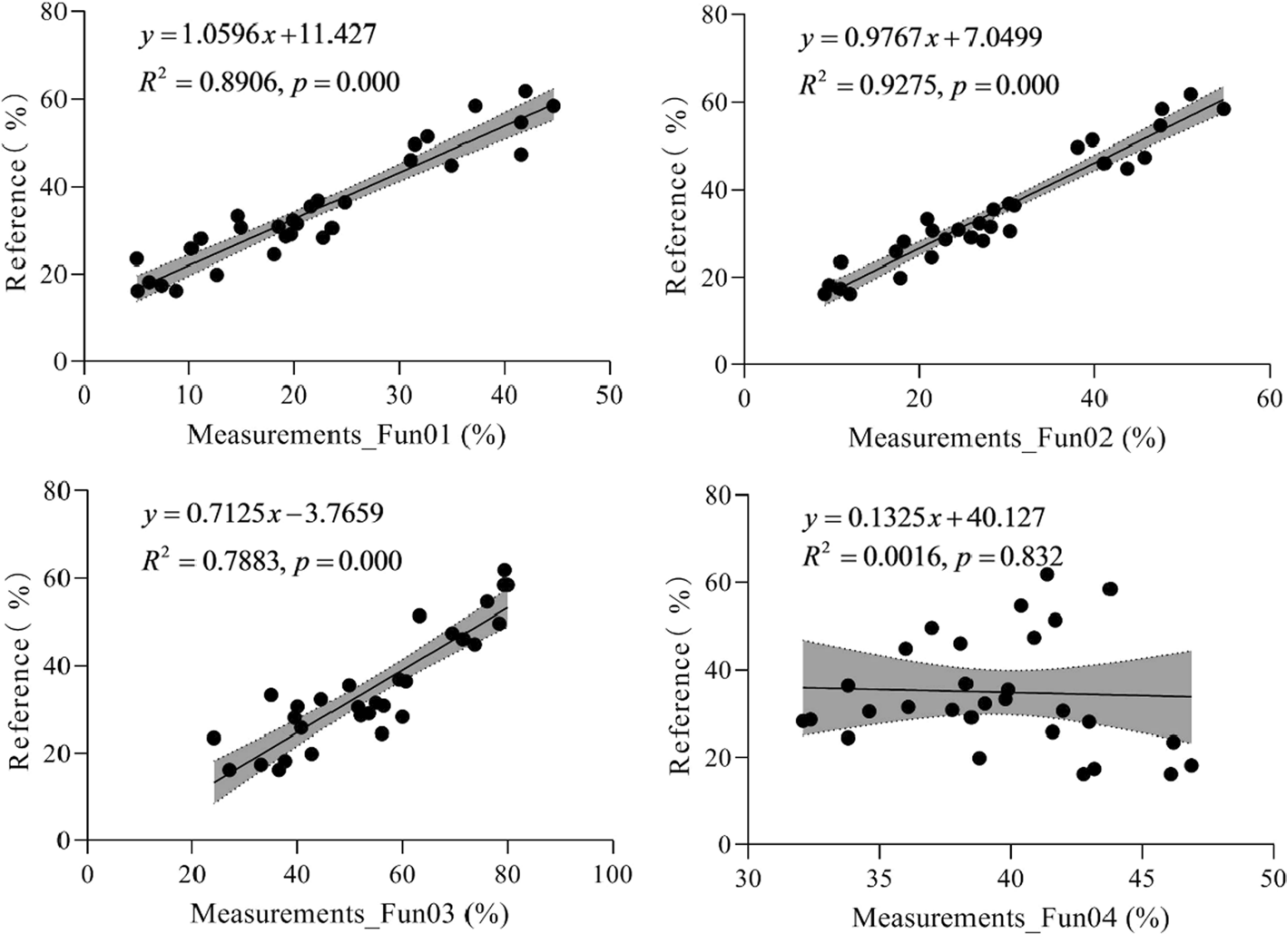
The linear relation between the fractional vegetation cover measured and the reference under the solar noon scenario. Shaded areas represent the 95% confidence intervals for the regression lines. Fun01 is based on the combination of $R/G$, $B/G$ and $2G-R-B$. Fun02 is the decision tree algorithm based on the RGB color. Fun03 uses the excess green index and the excess red index to identify the plants in the image. Fun04 uses the excess green index and the Otsu algorithm to identify the plants in the image

Figure 10 shows the linear relationship between the measured and reference FVC values. From Fig. 10, we can see that the R^2^ of Fun02 is higher than that of the other algorithms, followed by Fun01, Fun03, and Fun04.

Table 3 lists the accuracy variables for the different algorithms under the solar noon scenario and the accuracy of Fun02 is higher than those of the other algorithms.

**Table 3:** Measurement accuracy of different algorithms under the solar noon scenario

	MAPE (%)	BIAS	relBIAS (%)	RMSE	relRMSE(%)
Fun01	39.90	− 12.74	− 40.00	13.48	42.82
Fun02	20.60	− 6.39	− 20.67	7.30	24.49
Fun03	69.50	− 24.48	− 59.67	30.72	73.71
Fun04	47.00	4.77	33.00	14.65	69.04

Fun01 is based on the combination of $R/G$, $B/G$ and $2G-R-B$. Fun02 is the decision tree algorithm based on the RGB color. Fun03 uses the excess green and red indices to recognize the plants in the image. Fun04 uses the excess green index and the Otsu algorithm to identify the plants in the image

MAPE, mean absolute percentage error; relBIAS, relative bias; RMSE, root mean square error; relRMSE, relative root mean square error

Under the solar noon scenario, the accuracy of Fun02 was higher than that of the other algorithms. The mean RE was 20.6%, and Fun02 tended to underestimate the FVC in most measurements.

## Discussion

Fun01 is capable of identifying green plants with a high level of accuracy and efficiency [8]. In our study, under the scenario of overcast sky, the MAPE of the FVC measured using Fun01 was 8.68% (Table 1), while the MAPE increased to 47.8% (Table 2) under the solar forenoon scenario, and 39.9% (Table 3) under the solar noon scenario. The decrease in the measurement accuracy may be explained by the sunlit leaves being similar in color and brightness to the soil background under the solar forenoon and solar noon scenarios. These sunlit leaves may be misclassified as soil, resulting in a reduced level of plant identification accuracy. Under the scenarios of solar forenoon and solar noon, the shadow area in the image is large owing to the mutual occlusion of leaves and branches, and shaded leaves are easily misclassified as the background. However, these leaves and branches can be distinguished under the overcast sky scenario because they appear as real colors in an environment with scattered light. This could explain why the accuracy of Fun01 decreased abruptly under the solar forenoon and solar noon scenarios. This could also explain the underestimated FVC for Fun01 under the solar forenoon and solar noon scenarios. Based on this analysis, we conclude that Fun01 can be used to perform FVC measurements under the overcast sky scenario.

Fun02 is a decision tree algorithm based on the pixel DN of plants and the background components in the red, blue, and green channels. Zhang et al. [7] used this algorithm to estimate the FVC of *Festuca ovina* grassland and found that the RE of the FVC measured ranged from 9.63% to 0.08%, and the MAPE was 3.56%. However, the accuracy of Fun02 in our study was relatively low compared with that of Zhang et al. [7], particularly under the overcast sky scenario. We also found that the accuracy of Fun02 was higher than that of the other algorithms under the solar forenoon and solar noon scenarios (Tables 2, 3). Therefore, compared with other algorithms, Fun02 is more suitable for measuring the FVC on sunny days, although it considerably underestimated the FVC in most cases. Another advantage of Fun02 is that it can theoretically separate both green plants and colored flowers from images. Other algorithms can only identify green plants in the image and may be confused by the presence of flowers in the image, leading to an underestimation of the FVC.

Fun03 has been shown to have a high level accuracy in measuring FVC, and almost 90% of the plant pixels can be correctly differentiated from the background using this algorithm [27]. However, in our study, the accuracy of Fun03 under all the scenarios was lower than that reported by Meyer and Neto [28]. Myer and Neto [28] measured the FVC of crops instead of natural plants, where the color was homogeneous and the composition of the background including bare clay soil, weathered corn stalks, and fresh wheat straw was simple and uniform. In our study, the plants in the images acquired were relatively diverse and complicated in terms of the species and colors, and the background was a mixture of soil, small rocks, litter, and other objects. This may have resulted in the low accuracy of the Fun03 in our study.

Fun04 is based on $ExG$ and Otsu was used to acquire a threshold to separate the plants from non-plant pixels. Otsu is often used in image segmentation because of its simple algorithm, small number of calculations, and the high degree of automation. However, Otsu is highly sensitive to noise and only exhibits good segmentation performance on images with bimodal variance between classes. Meanwhile, vegetation images acquired from natural grassland are complicated, and cases with bimodal variance between the image classes do not always exist [11, 20, 31]. Meyer and Neto [28] found that the accuracy of $ExG + O{\text{tsu}}$ was approximately 50% in the case of a complicated photo background with soil-residue, and the accuracy reached 88% in the case of a simple background with bare soil. In our research, the accuracy of $ExG + O{\text{tsu}}$ was approximately 50% under the scenarios of overcast sky and solar noon (Tables 1, 3), which is similar to that of Meyer and Neto [28] for conditions with complicated backgrounds. We also found three abnormal values among the Fun04-measured FVC values (Fig. 4) under the overcast sky scenario. We inspected the three original images and found that light green lichens occupied a large area of the images. Fun04 classified these lichens as vegetation, whereas in the Photoshop-assisted identification method, the lichens were not identified as vegetation components. This led to three abnormal FVC values. If these three abnormal values had been omitted from the assessment, the accuracy of Fun04 would be similar to that of Fun01 under the scenario of an overcast sky.

The four algorithms selected in our study considered color as the only basis for discriminating plants from the background and calculating the FVC. These techniques make it difficult to effectively identify shaded and sunlit leaves [11]. To decrease the influence of shadows and sunlight on the measurement of FVC, Song et al. [6] introduced hue saturation intensity (HSI) to enhance the brightness of the shaded sections of the images. Coy et al. [21] used the Nelder–Mead algorithm to fit the distribution of vegetation and the background in the CIE Lab color space to determine a threshold for separating the vegetation from the background. However, these two algorithms were only applied to the FVC measurement of crops, and the robustness of these algorithms in the FVC measurement of natural vegetation needs to be validated. Sakamoto et al. [32] and Booth et al. [26] proposed that using artificial shelters to change the illumination conditions and reduce the contrast between the sunlit and shaded areas could avoid the shadow effect in small areas. This proposal may be an easy method to decrease the influence of shadows and sunlight on FVC measurements at the quadrat level (1 × 1 m). However, shading a large area is difficult in practice.

In recent years, the application of deep learning algorithms, such as convolutional neural networks, has improved the convenience and accuracy of image recognition and segmentation in different fields, such as face mask detection [33], classification of magnetic resonance images [34], X-ray images [35], and tree trunk identification [36]. The performance of deep learning has proven to be superior to other classical methods of computer vision [37]. Therefore, a deep-learning algorithm can potentially be used for image segmentation and cover estimation as the next step.

## Conclusions

Under the scenario of overcast sky, the algorithm based on the combination of $R/G$, $B/G$, and $E{\text{xG}}$(Fun01) presented the most accurate FVC measurement, although overestimation was observed for most measurements. Under the solar noon and the solar forenoon scenarios, the performance of the decision tree algorithm (Fun02) was superior to that of the other algorithms. However, it considerably underestimated the FVC in most cases. In the future, these two algorithms can be coded on a smart phone or pad, which allows researchers to easily acquire, process, and annotate digital images in the field to obtain real-time, georeferenced green canopy cover estimates.

## Supplementary Information

**Additional file 1.** Vegetation photoes acquired under the overcast sky, solar forenoon, and solar noon scenarios.

## Data Availability

The data are available upon reasonable request to the authors.

## References

[1] Purevdorj T, Tateishi R, Ishiyama T, Honda Y (2010). Relationships between percent vegetation cover and vegetation indices. Int J Remote Sens.

[2] Song YC (2001). Vegetation ecology.

[3] Sykes JM, Horril AD, Mountford MD (1983). Use of visual cover assessments as quantitative estimators of British woodland taxa. J Ecol.

[4] Chen ZG, Batunacun XZY, Hu YF (2014). Measuring grassland cover using digital camera images. Acta Prataculturae Sinica..

[5] Hahn I, Scheuring I (2003). The effect of measurement scales on estimating vegetation cover: a computer assisted experiment. Community Ecol.

[6] Song WJ, Mu XH, Yan GJ, Huang S (2015). Extracting the green fractional vegetation cover from digital images using a Shadow-Resistant Algorithm (SHAR-LABFVC). Remote Sens.

[7] Zhang CB, Li JL, Zhang Y, Zhou W, Qian YR, Yang F (2013). A quantitative analysis method for measuring grassland coverage based on RGB model. Acta Pratacul Sin.

[8] Patrignani A, Ochsner TE (2015). Canopeo: a powerful new tool for measuring fractional green canopy cover. Agron J.

[9] Graham EA, Yuen EM, Robertson GF, Kaiser WJ, Hamil Ton MP, Rundel PW (2009). Budburst and leaf area expansion measured with a novel mobile camera system and simple color thresholding. Environ Exp Bot..

[10] Richardson MD, Karcher DE, Patton AJ, McCalla JH (2010). Measurement of golf ball lie in various turfgrasses using digital image analysis. Crop Sci.

[11] Liu YK, Mu XH, Wang HX, Yan GJ (2012). A novel method for extracting green fractional vegetation cover from digital images. J Veg Sci.

[12] Kataoka T, Kaneko T, Okamoto H, Hata S. Crop growth estimation system using machine vision. In: Proceedings of the IEEE/ASME international conference on advanced intelligent mechatronics, Kobe, Japan, 20–24 July 2003; pp. b1079–b1083.

[13] Gée C, Bossu J, Jones G, Truchetet F (2008). Crop/weed discrimination in perspective agronomic images. Comput Electron Agric.

[14] Neto JC, Meyer GE, Jones DD (2006). Individual leaf extractions from young canopy images using Gustafson-Kessel clustering and a genetic algorithm. Comput Electron Agric.

[15] Kirci M, Gunes EO, Cakir Y, Senturk S. Vegetation measurement using image processing methods. In: Proceedings of the IEEE third international conference on agro-geoinformatics, Beijing, China, 11–14 August 2014; pp. 1–5.

[16] Bai XD, Li CN, Zhang XF, Wang Y, Cao ZG, Yu ZH (2013). Crop segmentation from images by morphology modeling in the CIE L*a*b* color space. Comput Electron Agric.

[17] Zhou Q, Robson M (2001). Automated rangeland vegetation cover and density estimation using ground digital images and a spectral-contextual classifier. Remote Sensing.

[18] Bawden O, Kulk J, Russell R, McCool C, English A, Dayoub F, Perez T (2017). Robot for plant species specific weed management. J Field Robot.

[19] Laliberte AS, Rango A, Herrick JE, Fredrickson EL, Burkett L (2007). An object-based image analysis approach for determining fractional cover of senescent and green vegetation with digital plot photography. J Arid Environ.

[20] Sadeghi-Tehran P, Virlet N, Sabermanesh K, Malcolm JH (2017). Multi-feature machine learning model for automatic segmentation of green fractional vegetation cover for high-throughput field phenotyping. Plant Methods.

[21] Coy A, Dale R, Michael T, David N, Jane C (2016). Increasing the accuracy and automation of fractional vegetation cover estimation from digital photographs. Remote Sens.

[22] Campbell JB (1996). Introduction to remote sensing.

[23] Lati RN, Filin S, Eizenberg H (2011). Robust methods for measurement of leaf-cover area and biomass from image data. Weed Sci.

[24] Lukina EV, Stone ML, Rann WR (1999). Estimating vegetation coverage in wheat using digital images. J Plant Nutr.

[25] Meyer GE, Hindman TW, Jones DD, Mortensen DA (2004). Digital camera operation and fuzzy logic classification of plant, soil, and residue color images. Appl Eng Agric.

[26] Booth DT, Cox SE, Louhaichi M, Johnson DE (2004). Technical note: lightweight camera stand for close-to-earth remote sensing. Rangel Ecol Manage.

[27] Paruelo JM, Lauenroth WK, Roset PA (2000). Technical note: estimating aboveground plant biomass using a photo-graphic technique. J Range Manage.

[28] Meyer GE, Neto JC (2008). Verification of color vegetation indices for automated crop imaging applications. Comput Electron Agric.

[29] Richardson AD, Jenkins JP, Braswell BH, Hollinger DY, Ollinger SV, Smith ML (2007). Use of digital webcam images to track spring green-up in a deciduous broadleaf forest. Oecologia.

[30] Otsu NA (1979). threshold selection method from gray-level histograms. IEEE Trans Syst Man Cybern.

[31] Macfarlane C, Ogden GN (2012). Automated estimation of foliage cover in forest understory from digital nadir images. Methods Ecol Evol.

[32] Sakamoto T, Shibayama M, Kimura A, Takada E (2011). Assessment of digital camera-derived vegetation indices in quantitative monitoring of seasonal rice growth. ISPRS J Photogramm Remote Sens.

[33] Kumar TA, Rajmohan R, Pavithra M, Ajagbe SA, Hodhod R, Gaber T (2022). Automatic face mask detection system in public transportation in smart cities using IoT and deep learning. Electronics.

[34] Ajagbe SA, Amuda KA, Oladipupo MA, Afe O, Okesola K (2021). Multi-classification of Alzheimer Disease on magnetic resonance images (MRI) using deep convolution neural network approaches. Int J Adv Comput Res.

[35] Awotunde JB, Ajagbe SA, Oladipupo MO, Awokola JA, Afolabi OS, Timothy MO, Oguns YJ. An Improved machine learnings diagnosis technique for COVID-19 pandemic using chest X-ray images. In: Florez H, Pollo-Cattaneo MF, editors. Applied informatics. ICAI 2021. Communications in computer and information science. 2021; 1455. Springer, Cham.

[36] Song CY, Yang B, Zhang L, Wu DX (2021). A handheld device for measuring the diameter at breast height of individual trees using laser ranging and deep-learning based image recognition. Plant Methods..

[37] Russakovsky O, Deng J, Su H, Krause J, Satheesh S, Ma S, Huang ZH, Karpathy A, Khosla A, Bernstein M, Berg AC, Fei-Fei L (2015). ImageNet large scale visual recognition challenge. Int J Comput Vis..

