# Supplementary material for: Adaptiveness of RGB-image derived algorithms in the measurement of fractional vegetation coverage
Source: BMC Bioinformatics. 2022 Aug 30;23:358. doi: 10.1186/s12859-022-04886-6 (PMC9429463; doi:10.1186/s12859-022-04886-6)
Supplement: Supplementary file 1 — Additional file 1. Vegetation photoes acquired under the overcast sky, solar forenoon, and solar noon scenarios. [file 12859_2022_4886_MOESM1_ESM.docx]

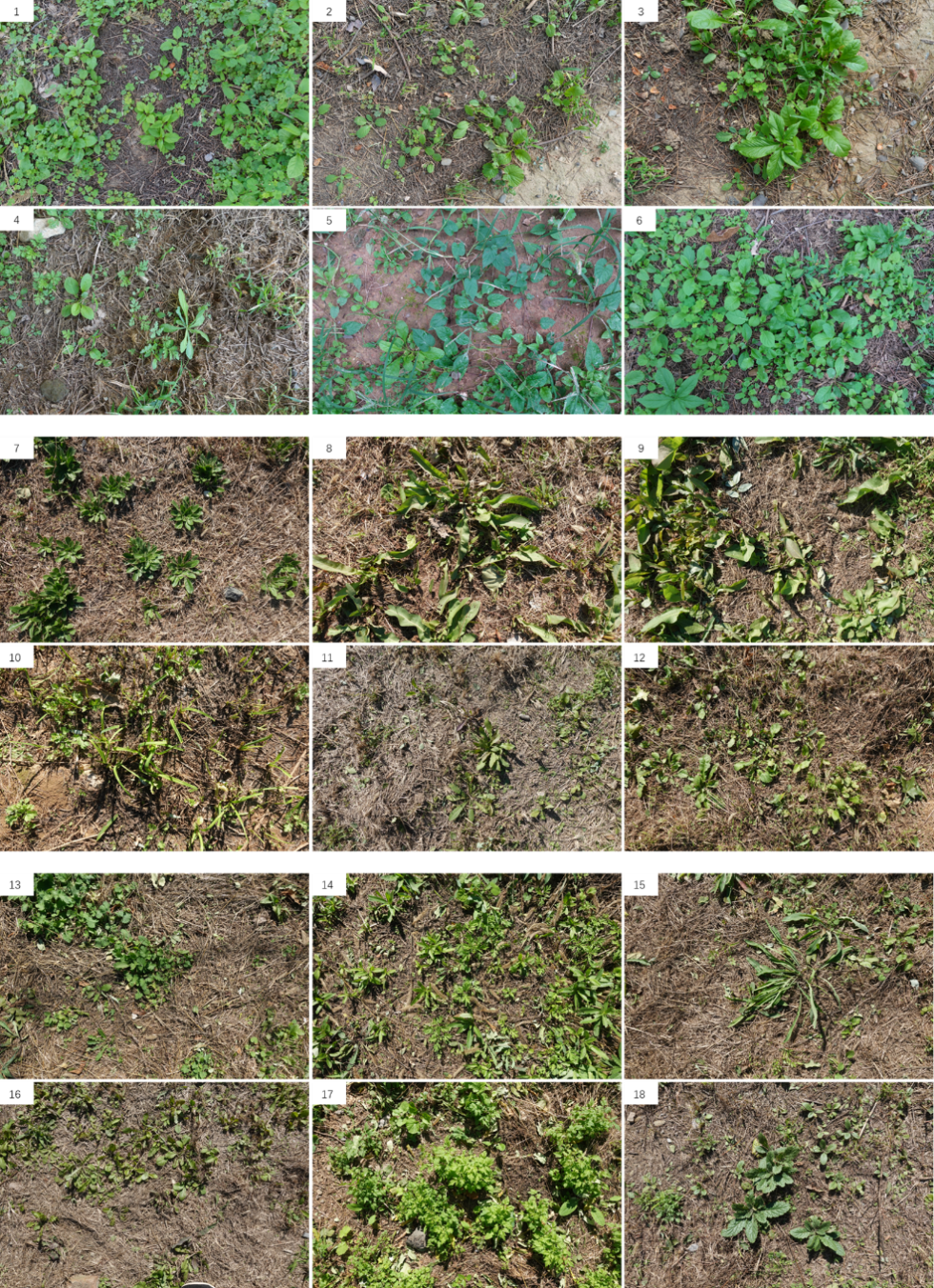


Fig.S1 Images acquired under different application scenarios

1-6 are images acquired under scenario overcast sky; 7-12 are images acquired under scenario solar forenoon; 13-18 are images acquired under scenario solar noon.
